# Sympatric Breeding Auks Shift between Dietary and Spatial Resource Partitioning across the Annual Cycle

**DOI:** 10.1371/journal.pone.0072987

**Published:** 2013-08-30

**Authors:** Jannie Fries Linnebjerg, Jérôme Fort, Tim Guilford, Anna Reuleaux, Anders Mosbech, Morten Frederiksen

**Affiliations:** 1 Department of Bioscience, Aarhus University, Roskilde, Denmark; 2 Department of Zoology, University of Oxford, Oxford, Oxfordshire, United Kingdom; 3 Arctic Research Centre (ARC), Aarhus University, Aarhus, Denmark; CNRS, Université de Bourgogne, France

## Abstract

When species competing for the same resources coexist, some segregation in the way they utilize those resources is expected. However, little is known about how closely related sympatric breeding species segregate outside the breeding season. We investigated the annual segregation of three closely related seabirds (razorbill *Alca* *torda* , common guillemot *Uria* *aalge* and Brünnich’s guillemot *U* *. lomvia* ) breeding at the same colony in Southwest Greenland. By combining GPS and geolocation (GLS) tracking with dive depth and stable isotope analyses, we compared spatial and dietary resource partitioning. During the breeding season, we found the three species to segregate in diet and/or dive depth, but less in foraging area. During both the post-breeding and pre-breeding periods, the three species had an increased overlap in diet, but were dispersed over a larger spatial scale. Dive depths were similar across the annual cycle, suggesting morphological adaptations fixed by evolution. Prey choice, on the other hand, seemed much more flexible and therefore more likely to be affected by the immediate presence of potential competitors.

## Introduction

Precisely how ecological communities consisting of competing species are able to maintain long-term biodiversity has been debated for decades [1]. Providing evidence for the various theories suggested is difficult, but there is no doubt that both intra- and interspecific competition are prevalent. However, it is often only possible to speculate to what extent the divergence seen between extant species is a result of current or past ecological competition driving morphological or behavioural specialization. Nevertheless, when closely related species coexist, resource partitioning is expected since interspecific competition is likely to be more pronounced due to their similar biology than among non-related species [2]. Resource partitioning may be expressed in, amongst other things, interspecific differences in prey, foraging habitats or foraging strategies [3–6]. The degree of resource partitioning is expected to vary between seasons, particularly for migrating species where spatial overlap can differ markedly across the annual cycle.

Seabirds provide an illustrative case, as they often breed highly concentrated in large multi-species colonies, and spend the rest of the year dispersed over large areas at sea. Whilst breeding, seabirds are central place foragers and their foraging behaviour is strongly affected by the need to return to land to reproduce [7,8]. In addition to the constraining influences of prey distribution and mobility, this type of foraging demand greatly restricts foraging options [9] and leads to increased energy expenditure. Both intra- and interspecific competition would thus be expected to be more intense within the breeding season than outside it. Furthermore, seabird colonies often consist of thousands of breeding pairs, resulting in increased potential for food competition [10]. As a result, much research has been conducted on how sympatric breeding seabirds limit competition during the breeding season by segregating spatially, temporally and/or in diet [11–13]. It has also been found that some seabirds not only segregate by species, but also by sex within each species [14–16]. Many previous studies were limited to one breeding season, corresponding to less than three months for many seabirds. Recently the use of miniature data-loggers and stable isotope techniques has made it possible to obtain detailed information about seabirds’ utilization of the marine environment and prey partitioning during the non-breeding season [17–20]. Despite this, to date only very few studies have combined the two techniques and the degree of spatial and dietary segregation outside the breeding season is still poorly known [21–23].

In this study we used geolocation (GLS, Global Location Sensing) and GPS (Global Positioning System) tracking technologies in combination with stable isotope analyses and dive depth analyses to examine the year-round resource partitioning among three closely related seabird species breeding sympatrically in Southwest Greenland. In the Sub-Arctic Atlantic Ocean, three auk species (razorbill ( *Alca* *torda* ), common guillemot ( *Uria* *aalge* ) and Brünnich’s guillemot ( *U* *. lomvia* )) often breed in mixed colonies. They are similar in size (razorbills being slightly smaller), occupy the same habitats and are all pursuit diving predators. This makes sympatric breeding auks excellent study organisms for investigating whether and how resource partitioning and segregation between species change over the annual cycle.

We hypothesized that as a consequence of sympatric colonial breeding followed by migration away from the colony, different spatial constraints might operate inside and outside the breeding season. Hence, we predicted that dietary segregation would be strongest during the breeding season, but spatial segregation more likely outside the breeding season when birds are away from the colony.

## Methods

### Study site

Fieldwork was carried out in July and August 2009, 2010 and 2011 at Kitsissut Avalliit (Southwest Greenland, 60°46’ N, 48°28’ W), the most southern major seabird colony in Greenland [24]. Kitsissut Avalliit holds the highest diversity of breeding seabirds in Greenland, with five sympatric breeding auks (razorbills, common guillemots, Brünnich’s guillemots, black guillemots *Cepphus* *grylle* and Atlantic puffins *Fratercula* *arctica* ), including the largest number of common guillemots known to breed in Greenland. Kitsissut Avalliit supports approx. 500 individuals of breeding razorbills [25], with a likely increase in numbers since the last count, and 3000 individuals of breeding common and Brünnich’s guillemots (common guillemots making up approx. 10% of the total number [26]).

The study was performed in accordance with the ethical guidelines promoted by the Association for the Study of Animal Behaviour. All handling of birds in this study was performed in the shade to avoid heat stress and the head of the bird was covered throughout in order to minimise stress. Birds were released after less than 15 minutes of handling. All necessary permits to conduct fieldwork at Kitsissut Avalliit as well as handling, ringing, deploying loggers and taking samples from the different species of birds were obtained from the Ministry of Fisheries, Hunting and Agriculture (APNN) in Greenland (Jr. Nr. 66.01.13 and Dok. Nr. 657095). No approval from an Ethics committee is required to conduct fieldwork and handling birds (ringing, sampling and deployment of loggers) in Greenland. However, as part of the approval process the Ministry of Fisheries, Agriculture and Hunting do take animal welfare into account. Our application thus contained information on fieldwork protocols (including blood sampling), and the permit was given conditional on us following these protocols.

### GPS data and processing

To map the spatial distribution of foraging during the three breeding seasons, we deployed GPS loggers on a total of seven razorbills, five common guillemots and six Brünnich’s guillemots. Three types of GPS loggers were used: i-gotU GT120 (Mobile Action Technology, Inc; removed from their housing; sampling rate = 10 min), TM-TAG (e-Shepherd Solutions, Guardbridge, UK; sampling rate = 2 min) and EP-3.1 (Ecotone, Poland; sampling rate = 2 min), recording date, time, longitude and latitude for approximately two days. GPS loggers were water-proofed using heat shrink tubing (Finishrink) and attached to the back feathers using Tesa® cloth tape, allowing loss of the logger through feather moult, or decay of the tape’s adhesive, in case of failed recapture. Total mass of GPS loggers was approx. 16 g (including attachment material), and corresponded to < 2.4% of adult body mass of the lightest bird. GPS data were processed in ArcGIS 10. Foraging areas were defined as areas where birds were diving and/or resting on water, therefore all points where birds were recorded in flight or at the colony were excluded.

### Geolocator data and processing

GLS loggers were used to map the overwintering areas of razorbills, common guillemots and Brünnich’s guillemots. GLS loggers use ambient light to estimate the timings of sunset and sunrise. The latitudinal position can then be estimated from day length, and longitudinal position from the time of local midday [27,28].

Two types of GLS loggers were used in this study: GLS-MK9 (British Antarctic Survey, U.K), and Lotek LAT2500 (Lotek Marine Technology, St. John’s, Newfoundland, Canada), both of which were attached to metal rings and deployed on the tarsus of the birds. The masses of GLS-MK9 and Lotek LAT2500 in air were approximately 5.3g and 6.2g respectively (including attachments), corresponding to < 1% of adult body mass of the lightest bird.

All birds were caught on the nest, weighed, and a total of 51 loggers were deployed on razorbills, common guillemots and Brünnich’s guillemots (18, 16 and 17 respectively). In total 33 loggers were retrieved, of which 23 provided data. Results were obtained from nine razorbills, eight common guillemots and six Brünnich’s guillemots. Seven birds were tracked for two years (two razorbills, three common guillemots and two Brünnich’s guillemots), thus our data represent 30 bird-years.

Upon recapture, birds were re-weighed and a blood sample of 50µl was taken from either the tarsus or brachial vein and stored in heparin coated vials for use in sex determination by DNA analysis.

Geolocator light data from BAS loggers were processed using the BASTrak software package. A light threshold of 10 and a sun angle of -3^°^ for razorbills and -3.5^°^ for common and Brünnich’s guillemots were used. Sun angles were selected by visually inspecting positions derived using a range of angles during periods when birds were presumed to be near the colony (May–June). Light data from Lotek loggers were processed with LAT Viewer Studio Software (Lotek Wireless, Newmarket, Ontario) using the template fitting method [29]. Data from each bird were processed individually and positions inspected visually. Data were subsequently filtered by removing the breeding period and autumn and spring equinoxes (app. 1-3 weeks on either side). All locations from GLS data were smoothed using a three-position moving average based on spherical trigonometry [30] to reduce the influence of outliers caused by the inherent error in light-based geolocation (mean error ca. 185 km [31]).

Kernel densities for GLS and GPS data were analysed in ArcView GIS 3.2 (ESRI) using the Animal movement extension. We used 50% and 95% kernel density contours to represent the core area of foraging activity and the area of active use, respectively [32]. We used least square cross validation (LSCV) and the ad hoc option in the Animal movement extension to estimate the smoothing factor for GLS and GPS positions respectively [33].

### Dive data

Vertical habitat use of birds was determined from diving information. In addition to geolocation, Lotek loggers recorded pressure throughout the year every sixth or tenth minute depending on the programming of the individual logger. None of the three species have been found to have dive durations of more than 5 min (summarized in Table 2 in [34,35]). Thus, each data point was considered as belonging to an individual dive and used to quantify dive depth during the non-breeding season. During the breeding season dive data were recorded by means of Lotek LAT2500 and LAT1500 time-depth recorders with a sampling rate of eight seconds during short-term deployments. These data were analysed using MultiTrace Dive (Jensen Software Systems) and maximum dive depth extracted. In order to exclude shallow dives during bathing and other non-foraging activities, as well as to account for inaccuracy of the depth recorders at 0 m, only dives deeper than four meters were considered. Non-breeding season dive data consisted of a single data point per dive and were thus not directly comparable with breeding season data. However, due to the typical U shape of auk dives, most recorded values were likely to represent the bottom phase of dives and therefore maximum dive depth.

### Stable isotope analysis (SIA)

To obtain information on trophic status across the annual cycle, stable isotope analyses were performed on three different tissues (blood, back feathers and throat feathers) collected from breeding adult birds during July 2011. The two batches of feathers were sampled according to the moulting sequence of the three auk species. Soon after breeding (in August–September), all species undergo a complete moult when cover and flight feathers are replaced within a few weeks, leaving the birds flightless. In the spring, they have a partial pre-breeding moult, when only face and throat feathers are replaced [36]. Though the exact time of this partial moult is unknown, face and throat feathers are replaced before the return to the breeding colony. In this study we therefore assume the spring moult to be happening in early to mid-April, as birds have been observed on the ledges in late April (25^th^ and onwards) in full breeding plumage. The different body tissues incorporate the isotopic signatures of resources at different rates (a few weeks for blood and the growing time for feathers [37–39]), and are therefore considered to reflect isotopic signatures for the following periods: the breeding season (blood), fall (September; back feathers) and spring (April; throat feathers).

Blood samples were taken from 14 razorbills, 13 common guillemots and 10 Brünnich’s guillemots and stored in 70% ethanol before preparation for stable isotope analysis. Prior to analysis, samples were dried for 48 h at 60^°^C and then homogenized. Feathers were collected from 15 razorbills, 13 common guillemots and 11 Brünnich’s guillemots, and stored in zip lock bags before analysis. Feathers were rinsed in a 2:1 chloroform: methanol solution, rinsed 2× in a methanol solution, dried for 48 h at 60°C and homogenized with scissors. Analyses were performed at the Biosystems Department (BIO-309) at Risø National Laboratory, Denmark on 1 mg subsamples of dried and homogenized tissues loaded into tin cups, using a CE 1110 elemental analyser (ThermoFinnigan, Milan, Italy) coupled in continuous flow mode to a Finnigan MAT, Delta PLUS isotope ratio mass spectrometer. Stable isotope abundances were expressed in δ notation as the deviation from standards in parts per thousand (‰) according to the following equation: δX = [(R_sample_/R_standard_)-1] × 1000, where X is ^13^C or ^15^N and R is the corresponding ratio ^13^C/^12^C or ^15^N/^14^N. The R_standard_ values were based on Vienna-PeeDee Belemnite (VPDB) for ^13^C and atmospheric N_2_ (air) for ^15^N. The δ^15^N isotopic values reflect the relative trophic position of birds, while the δ^13^C values reflect the source of carbon (allowing distinction between nearshore and pelagic foraging habitats [40]).

### Statistical analysis

We applied multivariate analysis of variance (MANOVA Pillai’s trace) to examine the isotopic data for differences between species. Each tissue type was analysed separately. The dependent variables were δ^15^N and δ^13^C with species and sex as fixed factor. If a significant result was found in the dependent variables we applied one-way analysis of variance (ANOVA) followed by post-hoc Tukey tests. We used linear mixed effect models (LME) with species as a fixed factor to examine species variation in dive depth. Non-independence of data within individuals was accounted for by including individual random effects. All data were transformed when necessary, to meet the requirements of normality. All analyses were carried out in R 2.15.0 (R Development Core Team 2012), and p < 0.05 was regarded as statistically significant.

## Results

### Breeding and non-breeding spatial distribution

#### Horizontal distribution

**Figure 1:**
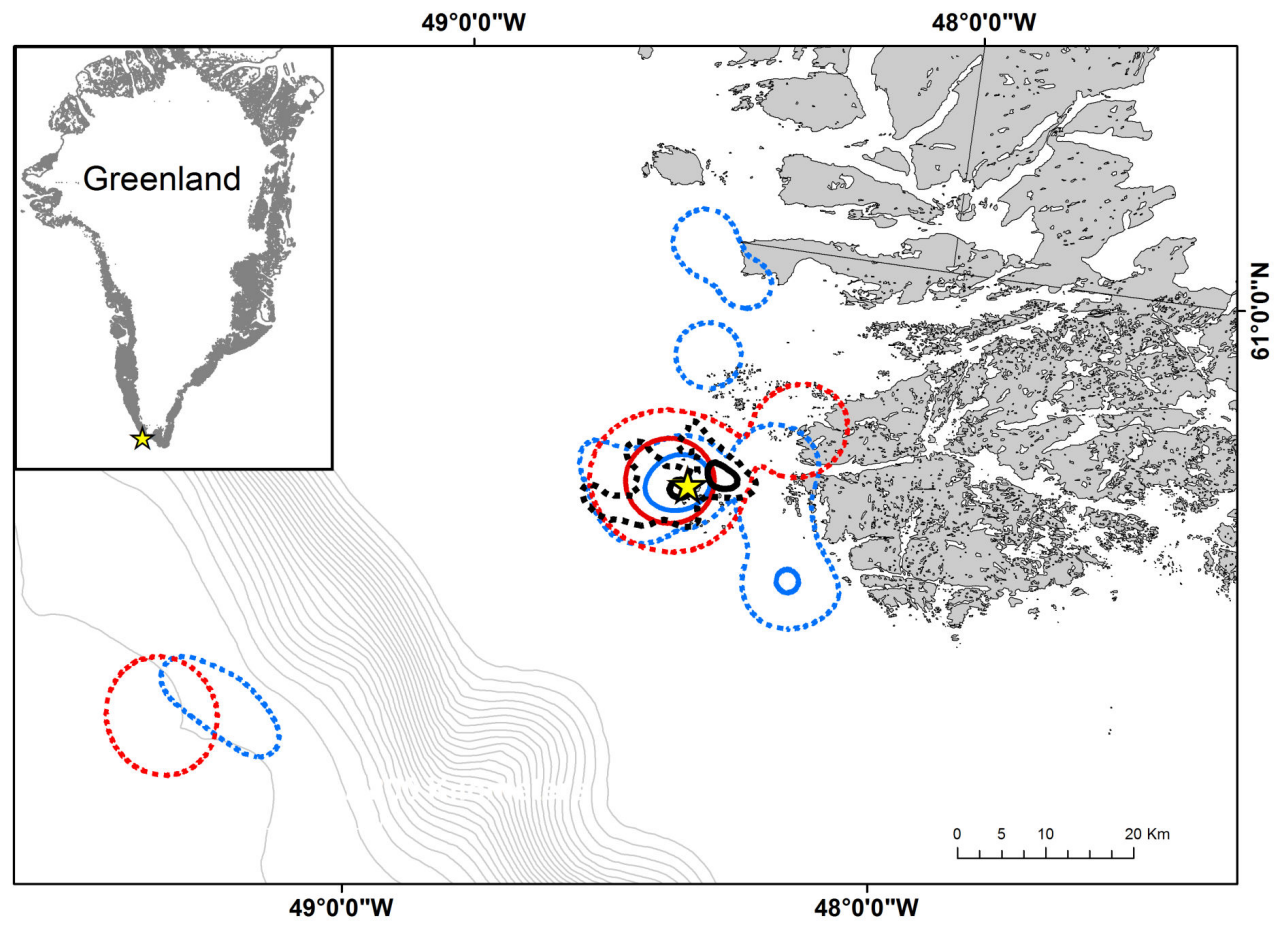
Spatial distribution of foraging auks during the breeding season at Kitsissut Avalliit, Greenland. Razorbills (n = 7) shown in blue, common guillemots (n = 5) in red and Brünnich’s guillemots (n = 6) in black. 50 and 95% kernel contours are represented by solid and dashed lines, respectively. The breeding colony is marked with a yellow star and differences in depth between isobaths are 100 m.

During the breeding season razorbills, common and Brünnich’s guillemots overlapped in their core foraging areas (50% kernel distribution, Figure 1). While most foraging occurred within 20 km of the colony, razorbills and common guillemots made occasional trips to the continental shelf edge approximately 60 km from the colony. Both razorbills and common guillemots foraged closer to the mainland than Brünnich’s guillemots (95% kernel distribution, Figure 1).

**Figure 2:**
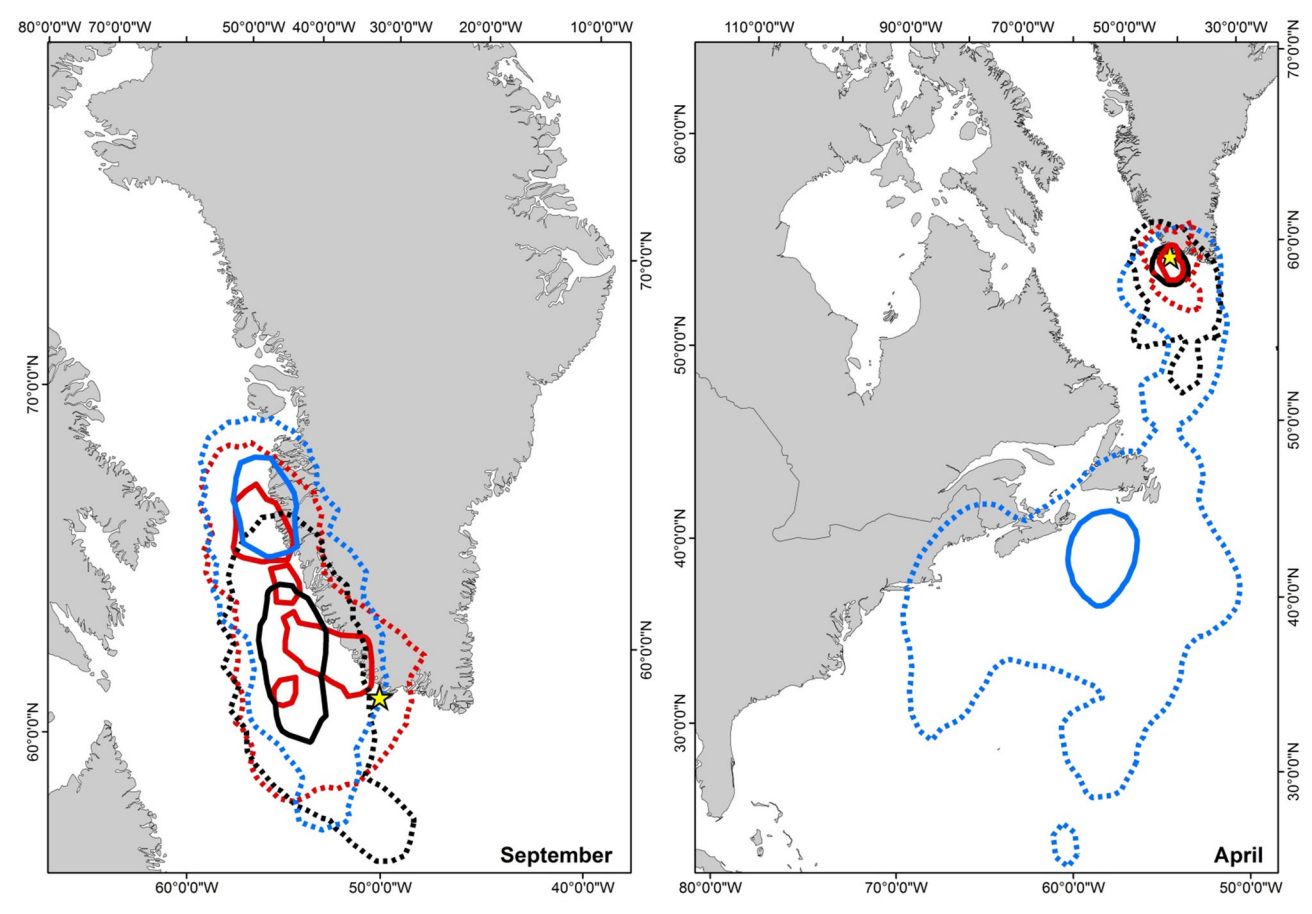
Spatial distribution of auks during September and April. 50% (solid) and 95% (dashed) kernel distribution of razorbills (n = 9, blue), common guillemots (n = 8, red) and Brünnich’s guillemots (n = 6, black) during the first half of September (left panel) and April (right panel). Razorbills left the east coast of North America at the end of April, thus the activity area north of 50^°^ N represents the period from 24^th^ of April and onwards. The breeding colony is marked with a yellow star.

During the first half of September, all three species had overlapping activity areas (95% kernel distribution), but where common guillemots had overlapping core foraging areas with both razorbills and Brünnich’s guillemots, razorbills and Brünnich’s guillemots did not overlap in their core area (50% kernels) (Figure 2 left panel). In general Brünnich’s guillemots had a more southerly overall distribution in September than both razorbills and common guillemots (Figure 2 left panel).

All three species left the breeding colony in mid-August and most individuals moved north along the West Greenland coast (Figure S1). After the initial northward movement, the razorbills migrated to waters off Atlantic Canada and USA in October. All razorbills left their overwintering areas in late April (24^th^ and onwards) and were back at the breeding site in late April/early May. Most common guillemots wintered just off the coast of west Greenland, but one individual spent February and March off the east coast of Greenland (Figure S1). All but one Brünnich’s guillemot wintered off the west Greenland coast. Both guillemot species arrived back at the breeding ledges in late April.

During the pre-breeding period (April), razorbills were spatially segregated from the two guillemot species until the 24^th^ of April and onwards (Figure 2, right panel). Brünnich’s guillemots and common guillemots, on the other hand, overlapped spatially, but with Brünnich’s guillemots having a larger, more southerly distribution than the common guillemots.

#### Vertical distribution

**Figure 3:**
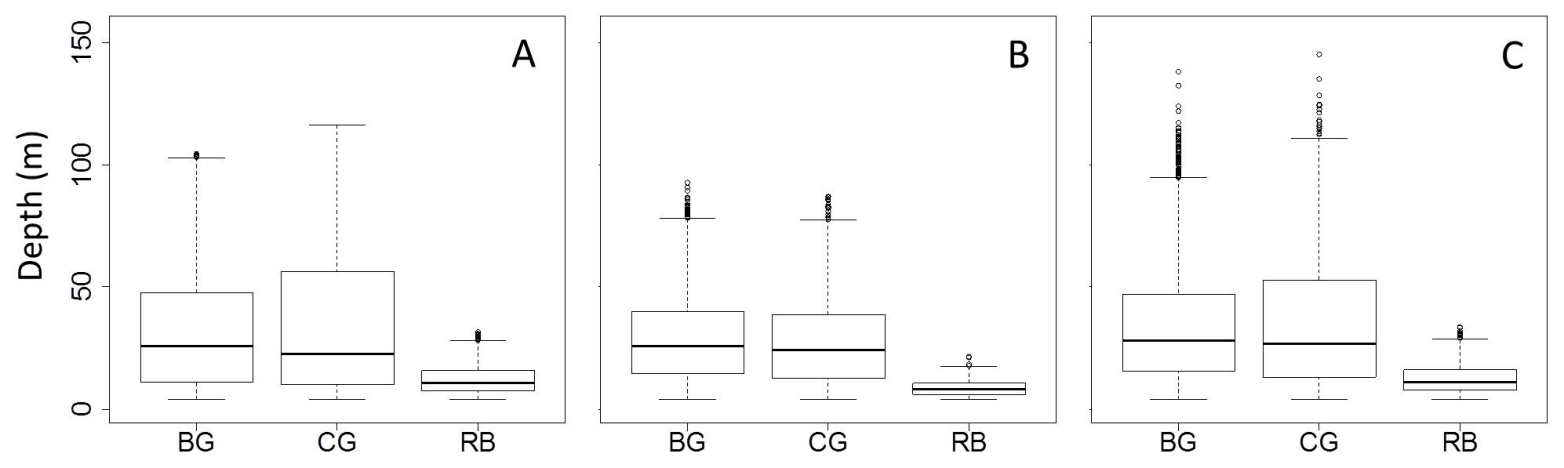
Median maximum dive depth of auks on an annual cycle. Median maximum dive depth of Brünnich’s guillemot (BC), common guillemots (CG) and razorbills (RB) during the breeding season (A), post-breeding period (September, B) and pre-breeding period (April, C). The bottom and top of the box show the 25^th^ and 75^th^ percentiles, respectively around the median (solid line). The vertical dashed lines (whiskers) show either the maximum value or 1.5 times the difference in the response variable between its first and third quartiles, which ever is the smallest. Outliers are shown with circles. When there are no outliers the whiskers show the maximum and minimum values.

During the breeding season, we found a clear difference in dive depth between razorbills (mean = 12.1 m) and the two guillemot species (Table 1, Figure 3A), but not between Brünnich’s guillemots and common guillemots (mean dive depth 33.1 m and 35.5 m respectively, Table 1, Figure 3A).

**Table 1:** Summary table of spatial (horizontal and vertical) and trophic partitioning between razorbills, common guillemots and Brünnich’s guillemots during the breeding, post-breeding and pre-breeding periods.

		**Razorbills vs. common guillemots**	**Razorbills vs. Brünnich’s guillemots**	**Common guillemots vs. Brünnich’s guillemots**
**Breeding (July–Aug)**	**Horizontal**	Overlapping core and activity area	Overlapping core and activity area	Overlapping core and activity area
	**Vertical**	p = 0.0031	p = 0.0008	p = 0.2812
	**Trophic**	δ13C: p = 0.73	δ13C: p < 0.0001	δ13C: p < 0.0001
		δ^15^N: p = 0.32	δ^15^N: p = 0.081	δ^15^N: p = 0.003
**Post-breeding (Sep)**	**Horizontal**	Overlapping core and activity area	Non-overlapping core area. Overlapping activity area	Overlapping core and activity area
	**Vertical**	p < 0.0001	p < 0.0001	p = 0.7909
	**Trophic**	δ13C: p = 0.56	δ13C: p = 0.004	δ13C: p = 0.05
		δ^15^N: p = 0.83	δ^15^N: p = 0.63	δ^15^N: p = 0.93
**Pre-breeding (April)**	**Horizontal**	Non-overlapping core area. Overlapping activity area from 24th of April and onwards.	Non-overlapping core area. Overlapping activity area from 24th of April and onwards.	Overlapping core and activity area
	**Vertical**	p = 0.004	p = 0.004	p = 0.92
	**Trophic**	δ13C: p = 0.42	δ13C: p = 0.74	δ13C: p = 0.89
		δ^15^N: p = 0.028	δ^15^N: p = 0.16	δ^15^N: p = 0.78

P-values for vertical and trophic partitioning are derived from linear mixed effect models (LME) and post-hoc Tukey tests, respectively. p < 0.05 is regarded as statistically significant.

There was a clear difference in dive depth during the post-breeding season (September) between razorbills (mean = 8.6 m) and both Brünnich’s guillemots and common guillemots (Table 1, Figure 3B) but not between Brünnich’s guillemots and common guillemots (mean = 28.7 m and 27.4 m, respectively, Table 1, Figure 3B).

During the pre-breeding season (April), there was again a clear difference in dive depth between razorbills (mean = 12.4 m) and the two guillemot species (Table 1, Figure 3C), but again no clear difference in dive depth between the Brünnich’s guillemot and common guillemot (mean = 34.4 m and 35.9 m respectively, Table 1, Figure 3C).

#### Trophic status

We did not detect any sex difference in the overall isotopic signature during the breeding, post-breeding or pre-breeding season for common guillemot (MANOVA, F_4,20_ = 1.454, p = 0.253, F_4,20_ = 1.429, p = 0.200 and F_4,20_ = 1.282, p = 0.310 respectively) and Brünnich’s guillemot (F_2,7_ = 1.550, p = 0.277, F_2,7_ = 0.2409, p = 0.791 and F_2,8_ = 0.300, p = 0.748 respectively). For razorbills, we did not detect any sex difference in the overall isotopic signatures during the breeding and pre-breeding season (MANOVA, F_4,22_ = 0.309, p = 0.868 and F_4,24_ = 0.612, p = 0.658 respectively), but detected a significant difference in the overall signature during the post-breeding season (F_4,22_ = 8.459, p = 0.000). We therefore tested for between-sex differences separately for δ^13^C and δ^15^N, and found a difference in δ^15^N between the sexes (ANOVA, p = 0.104 and p = 0.012 respectively). Despite the difference in δ^15^N for razorbills during the post-breeding season, we decided to pool the sexes in the comparisons of diet of all three species due to the small sample sizes in each season.

**Figure 4:**
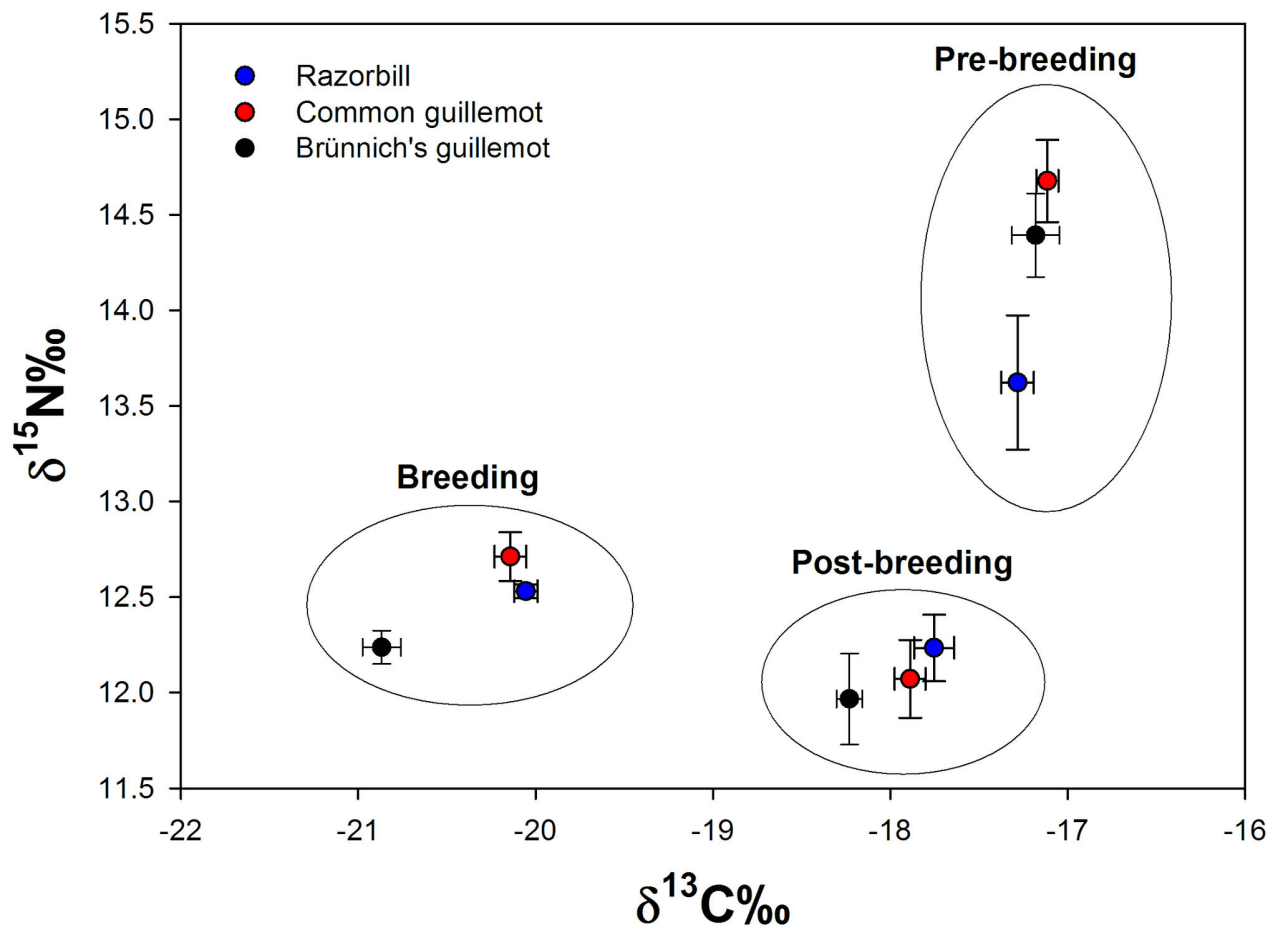
Stable isotope values of auks on an annual cycle. δ^13^C and δ^15^N values (mean ± SE) of blood (breeding), back-feathers (post-breeding) and throat feathers (pre-breeding), of adult razorbills (blue), common guillemots (red) and Brünnich’s guillemots (black).

During the breeding season, no difference was found between razorbills and common guillemots in δ^13^C or δ^15^N (Figure 4, Table 1), but Brünnich’s guillemots had lower values of both δ^13^C and δ^15^N compared to common guillemots and in δ^13^C compared to razorbills (Figure 4, Table 1). This suggests that during the breeding season, Brünnich’s guillemots were feeding at a lower trophic level and on a more pelagic diet than the other two species.

No difference was found during the post-breeding season between razorbills and common guillemots in δ^13^C (Figure 4, Table 1), but Brünnich’s guillemots had lower δ^13^C values compared to both razorbills and common guillemots (Figure 4, Table 1) indicating a more pelagic diet. There was no difference in δ^15^N between the three species during the post breeding season.

During the pre-breeding season, no difference in δ^13^C was found between the three species (Figure 4, Table 1). We found no difference in δ^15^N between Brünnich’s guillemots and both razorbills and common guillemots (Figure 4, Table 1), but razorbills differed from common guillemots in δ^15^N (Figure 4, Table 1).

## Discussion

By combining GPS and GLS spatial data with dive depth and stable isotope analysis, this study presents novel information on the dietary and spatial partitioning during the entire annual cycle of three closely related sympatric breeding auks. In accordance with our predictions, we demonstrate that during the breeding season, razorbills, common guillemots and Brünnich’s guillemots partitioned in either dive depth, diet or both, presumably due to the presence of potential competitors. During both the post-breeding and pre-breeding periods, the three species had an increased overlap in their isotopic niche, and presumably their diet. In addition, razorbill distribution during post-breeding and pre-breeding did not overlap with either of the guillemot species (Figure 2 [26,41,42]). Thus, the three species segregate in different dimensions during the breeding period compared to the two other time periods.

### Annual segregation

During the breeding season, we found a strong overlap in the core foraging areas (50% kernel contours) used by razorbills, common guillemots and Brünnich’s guillemots, although razorbills and common guillemots were found to forage closer to shore than Brünnich’s guillemots. This contrasts with previous studies which showed a segregation in foraging areas of sympatric breeding auks [43]. Moreover, Brünnich’s guillemots have, during the incubation period, been found to forage approximately 60 km from the breeding colony at Kitsissut Avalliit [26], thus the absence of foraging locations further away from the colony during chick rearing was interesting. Further studies should be conducted over several years during both incubating and chick-rearing periods to confirm that sympatric breeding auks in Southwest Greenland do not segregate in foraging areas.

Support for prey partitioning during the breeding season comes from the isotopic signatures, where differences in δ^15^N and δ^13^C indicated a strong segregation in diet between Brünnich’s guillemots and both razorbills and common guillemots (Figure 4). This is consistent with previous studies, where invertebrates have been found to play a more important part in the diet of Brünnich’s guillemots than common guillemots [44,45]. Less is known about the diet of razorbills, but it has been found to consist mainly of either crustaceans or fish depending on the geographic area and season [44,46]. In our study, stable isotope analyses indicated that breeding razorbills fed on a diet similar to common guillemots. Since common guillemots principally forage on fish throughout the year [44], this suggests that razorbills have a more fish-based diet at Kitsissut Avalliit. Razorbills and common guillemots, which used similar spatial areas, partitioned resources by segregating vertically, with razorbills diving significantly shallower than common guillemots.

An increase in the abundance of resources would be expected to result in a reduction in competition and a subsequent increase in resource overlap, since food partitioning is considered as a mechanism preventing food shortage [1]. This has for instance been found for common and Brünnich’s guillemots during the incubating period, where the two species were more segregated in their trophic level in food-limited years [47]. The abundance of food at our study site is unknown and our results only cover one breeding season. It is thus not possible to determine whether the segregation found was a result of food scarcity. Analyses of time budgets [26], however, do not indicate food scarcity during the breeding period. An alternative explanation to competition pressure could be that due to the constraints experienced during breeding, birds are simply feeding on the prey they are best adapted to exploit, and that this results in partitioning in diet during the breeding season.

Outside the breeding season, as expected, we found the three species to be distributed on a larger spatial scale and show a reduced segregation in trophic level. During the post-breeding period, Brünnich’s guillemots had slightly lower δ^13^C values indicating a more pelagic distribution. The core distribution area for razorbills and partly for common guillemots as well as the 95% kernel distribution suggests an earlier departure from the breeding area compared to Brünnich’s guillemots. Desynchronized departure from the breeding colony by different species has also been found elsewhere [48,49], and could be a way to segregate foraging areas during a period of reduced mobility when adults are flightless and males accompany and feed chicks at sea. Segregation of foraging areas has also been suggested to facilitate the coexistence of simultaneously moulting petrels, thus reducing competition [50].

During the period November through most of April, razorbills and the two guillemot species were completely segregated spatially, whereas common guillemots and Brünnich’s guillemots overlapped in their spatial distribution (Figure 2 [26]). This confirms previous studies which showed an increase the spatial segregation outside the breeding season of sympatric seabirds [19]. Due to the low accuracy of GLS loggers, small spatial scale segregation during the non-breeding season might be overlooked. However, we found no differences in isotopic signature or dive depth between the two guillemot species, suggesting that the inter-specific competition pressure is reduced during the pre-breeding period. In addition to spatial segregation, this could also be linked to the greater mobility and presumably lower density of birds at this time of year due to the absence of the central-place foraging constraint, which should reduce the likelihood of inter- and intraspecific competition [10].

Previous studies have shown that Brünnich’s guillemots shift their diet to a lower trophic level during late winter compared to immediately after breeding [51,52]. We found the opposite pattern, suggesting a more fish-based diet during the pre-breeding period. δ^13^C values in all three species increased over winter (from September to April), indicating a possible shift in foraging location from offshore to nearshore. As razorbills have different wintering grounds to the two guillemot species, the difference seen in δ^15^N might be the result of different isotopic signatures in the wintering areas rather than trophic level [53].

### Migration

Tracking razorbills, common and Brünnich’s guillemots breeding in southern Greenland with GLS loggers provided new insight into the ecology and non-breeding movements of these sympatric breeding auks. Immediately after the breeding season, birds migrated north as far as Disko Bay (approx. 69^°^ N), following the West Greenland Current (Figure S1). This is consistent with migration patterns being mainly determined by prevailing surface currents, which is expected during the flightless period. This northward migration has also been observed at a colony further north in West Greenland [54], although on a much smaller scale.

We found two distinct overwintering areas. One area, exclusively used by razorbills, was situated off the eastern coast of North America. This area is also extensively used by many other seabird species during the non-breeding season, not only birds breeding in North America but also from Europe [30,42,55,56]. Nonetheless, few seabirds breeding in the Atlantic Subarctic migrate as far south as waters off Cape Hatteras (approx. 35° N). The second area was off the coast of south-western Greenland, and was used exclusively by the two guillemot species. Waters offshore Southwest Greenland are also recognized as an important overwintering area for millions of birds breeding, not only locally, but also in e.g. Arctic Canada, Svalbard, Russia and Europe [57–59]. Although ringing recoveries suggest that some Brünnich’s guillemots from Kitsissut Avalliit overwinter off Newfoundland [60], none of the birds in this study spent the non-breeding season in that area.

It is very interesting that of the three species, the razorbill was the only one to leave Greenlandic waters, although in Europe razorbills are also known to migrate further south than common guillemots [61,62]. Possible explanations for this divergence of migratory and overwintering strategies include potential past competition and different post-glacial re-colonization routes.

## Conclusions

To our knowledge, this study is the first to reveal patterns of resource partitioning among three closely related sympatric breeding auks during an entire annual cycle. This study gives insight into how closely-related sympatric breeding seabirds partition resources during the breeding season when they are constrained by the need to return to the breeding site to feed their chicks; and how this resource partition is reduced in the non-breeding season due to the ability to move more freely and thereby segregate spatially. We found dive depth to be very similar across the annual cycle, indicating considerable conservatism, probably due to morphological adaptations to deep diving. Prey choice on the other hand seemed much more flexible and thus is more likely to be affected by the presence of potential competitors. In general, morphological and physiological traits are less plastic and evolve much more slowly than behavioural traits [63], and behavioural adaptation is therefore the most likely immediate ecological as well as evolutionary response to environmental change [64].

This study was conducted at a small colony in Southwest Greenland, where breeding season competition probably is not very intense; we suggest that additional studies are conducted at larger colonies where competition is more pronounced.

## Supporting Information

Figure S1 **Spatial distribution of auks on an annual cycle.** Distribution of razorbills (n = 8, blue), common guillemots (n = 8, red) and Brünnich’s guillemots (n = 6, black) during the non-breeding season in 2009/2010 and 2010/2011 based on GLS data. The panels show the monthly median position of each bird in August through to April.
(TIF)
