# Supplementary figures and images for: Sympatric Breeding Auks Shift between Dietary and Spatial Resource Partitioning across the Annual Cycle
Source: PLoS One. 2013 Aug 30;8(8):e72987. doi: 10.1371/journal.pone.0072987 (PMC3758292; doi:10.1371/journal.pone.0072987)

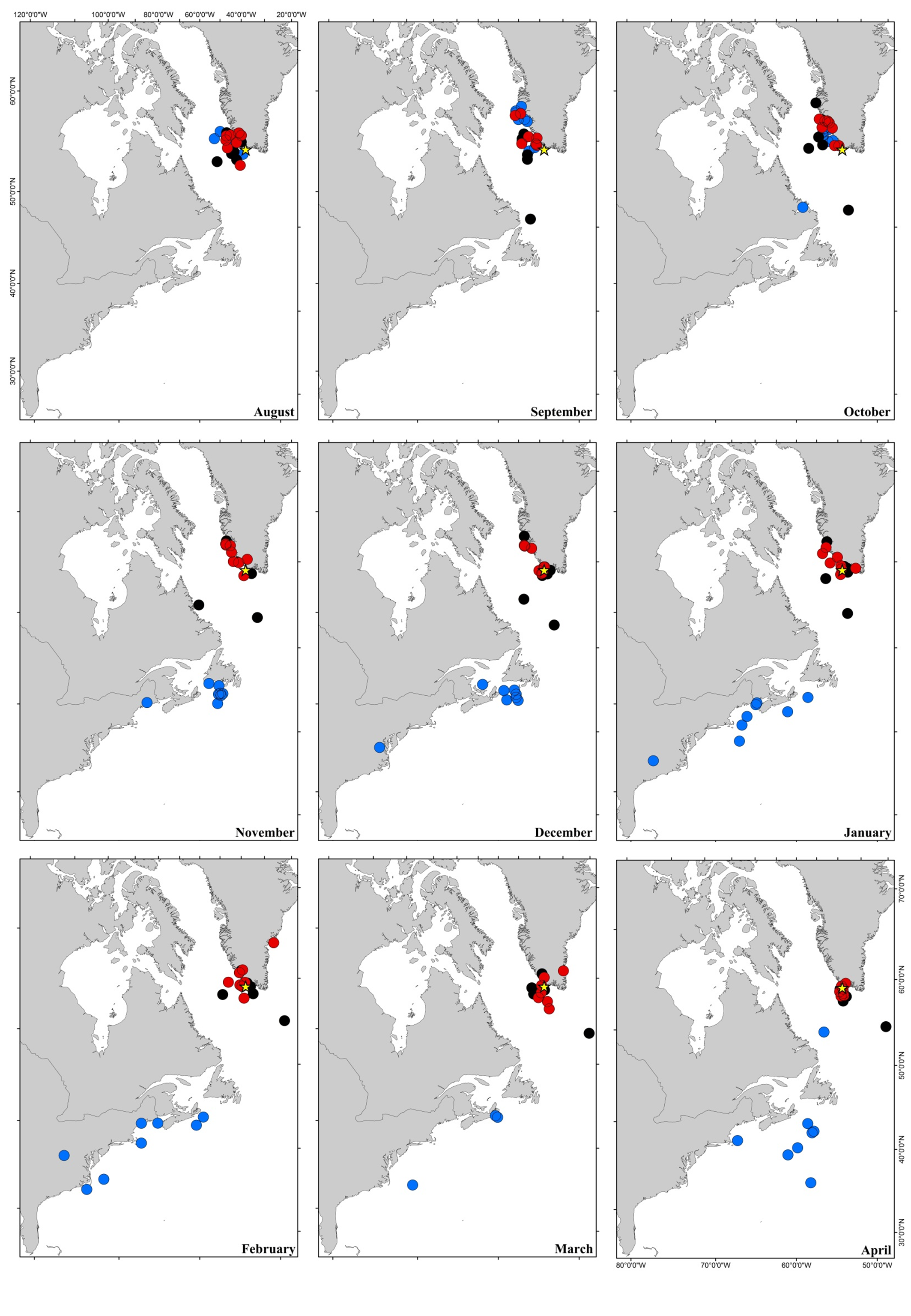

Supplement: Figure S1 — Spatial distribution of auks on an annual cycle. Distribution of razorbills (n = 8, blue), common guillemots (n = 8, red) and Brünnich’s guillemots (n = 6, black) during the non-breeding season in 2009/2010 and 2010/2011 based on GLS data. The panels show the monthly median position of each bird in August through to April. (TIF) [file pone.0072987.s001.tif]
